# Preference, satisfaction and critical errors with Genuair and Breezhaler inhalers in patients with COPD: a randomised, cross-over, multicentre study

**DOI:** 10.1038/npjpcrm.2015.18

**Published:** 2015-04-30

**Authors:** Sergi Pascual, Jan Feimer, Anthony De Soyza, Jaume Sauleda Roig, John Haughney, Laura Padullés, Beatriz Seoane, Ludmyla Rekeda, Anna Ribera, Henry Chrystyn

**Affiliations:** 1 Hospital del Mar-IMIM, CIBERES, Barcelona, Spain; 2 Pneumologie Odeonsplatz, Munich, Germany; 3 Institute of Cellular Medicine, Newcastle University, Newcastle upon Tyne, UK; 4 Department of Respiratory Medicine, Freeman Hospital, Newcastle upon Tyne, UK; 5 Servicio de Neumología, Hospital Universitari Son Espases, Palma de Mallorca, Spain; 6 Institut d’Investigació Sanitària de Palma (IdISPa), Palma de Mallorca, Spain; 7 CIBERES, Instituto Carlos III, Madrid, Spain; 8 Institute of Applied Health Sciences, University of Aberdeen, Aberdeen, UK; 9 Medical Affairs, Almirall, Barcelona, Spain; 10 R&D Centre, AstraZeneca, Barcelona, Spain; 11 Formerly of Almirall S.A., Barcelona, Spain; 12 Forest Research Institute, a subsidiary of Actavis PLC, Jersey City, NJ, USA; 13 Department of Pharmacy, University of Huddersfield, Huddersfield, UK

## Abstract

**Background:**

The specific attributes of inhaler devices can influence patient use, satisfaction and treatment compliance, and may ultimately impact on clinical outcomes in patients with chronic obstructive pulmonary disease (COPD).

**Aims:**

To assess patient preference, satisfaction and critical inhaler technique errors with Genuair (a multidose inhaler) and Breezhaler (a single-dose inhaler) after 2 weeks of daily use.

**Methods:**

Patients with COPD and moderate to severe airflow obstruction were randomised in a cross-over, open-label, multicentre study to consecutive once-daily inhalations of placebo via Genuair and Breezhaler, in addition to current COPD medication. The primary end point was the proportion of patients who preferred Genuair versus Breezhaler after 2 weeks (Patient Satisfaction and Preference Questionnaire). Other end points included overall satisfaction and correct use of the inhalers after 2 weeks, and willingness to continue with each device.

**Results:**

Of the 128 patients enrolled, 127 were included in the safety population (male *n*=91; mean age 67.6 years). Of the 110 of the 123 patients in the intent-to-treat population who indicated an inhaler preference, statistically significantly more patients preferred Genuair than Breezhaler (72.7 vs. 27.3%; *P*<0.001). Mean overall satisfaction scores were also greater for Genuair than for Breezhaler (5.9 vs. 5.3, respectively; *P*<0.001). After 2 weeks, there was no statistically significant difference in the number of patients who made ⩾1 critical inhaler technique error with Breezhaler than with Genuair (7.3 vs. 3.3%, respectively).

**Conclusions:**

Patient overall preference and satisfaction was significantly higher with Genuair compared with Breezhaler. The proportion of patients making critical inhaler technique errors was low with Genuair and Breezhaler.

## Introduction

There are numerous inhaler devices available for the delivery of bronchodilator treatment in patients with chronic obstructive pulmonary disease (COPD): for example, dry-powder inhalers (DPIs), pressurised metered-dose inhalers, soft mist inhalers and nebulisers.^1–3^ Each inhaler has advantages and disadvantages that may impact on a patient’s use, satisfaction and compliance with therapy, and ultimately affect clinical outcomes.^4,5^ Choosing the most appropriate inhaler for a patient is likely to positively influence their attitude to COPD, improve adherence to therapy and, consequently, have therapeutic benefits.^4^ It is therefore important that each patient be prescribed an inhaler device that they are both willing and able to use.^6^ This may be particularly true in elderly patients, who may have cognitive and/or physical issues that make inhaler use particularly challenging.^7^

Despite the importance of inhaled therapies in the management of COPD, incorrect inhalation technique is common among patients with COPD^8–10^ and is thought to be a key reason for reduced disease control in COPD.^11,12^ Issues relating to incorrect inhaler technique may also be worsened because many patients with COPD require multiple inhaled therapies (e.g., short-acting bronchodilators, long-acting bronchodilators, inhaled corticosteroids), which are often administered via separate inhalers that require distinct inhalation techniques for optimal use.^5,8,13^ Ensuring that patients with COPD always use the correct technique is key to achieving maximal benefit from inhaled therapies.^14^

The specific attributes of an inhaler device that may help to promote adherence and use of the correct technique include convenience, efficiency and ease of use, with simple instructions and minimal potential for errors.^3^ Genuair (Pressair in the United States; AstraZeneca, Mölndal, Sweden; registered trademarks of AstraZeneca A.B., Mölndal, Sweden, for use within the USA as Pressair and Genuair within all other licensed territories), is a novel, multidose, breath-actuated DPI for the delivery of aclidinium bromide (a long-acting muscarinic antagonist) either alone or in combination with formoterol fumarate (a long-acting β_2_-agonist), indicated as maintenance bronchodilator treatment in COPD.^15–17^ The objective of this study was to assess patient preference, satisfaction and critical inhaler technique errors after a 2-week period of inhaling placebo via Genuair and Breezhaler (Novartis, A.G., Basel, Switzerland). In contrast with Genuair, Breezhaler is a single-dose, breath-actuated DPI that works through the release of dry powder from a pierced gelatin capsule. The patient is required to load each capsule into the device before inhalation of a dose. Breezhaler was chosen as the comparator device in this study, as both inhalers are used in patients with COPD and contain a long-acting bronchodilator.

## Methods

### Study design and patients

This was a randomised, cross-over, open-label study performed in five centres in Germany, Spain and the United Kingdom between July and October 2013 (ClinicalTrials.gov registration: NCT01915784). The study was conducted in accordance with the ethical principles of the Declaration of Helsinki and the International Conference on Harmonisation Good Clinical Practice guidelines. The study complied with all applicable local regulatory requirements and was approved by the relevant local independent ethics committees. Each patient provided written informed consent prior to participation in the study.

Male or female patients were eligible for inclusion in the study if they were aged ⩾40 years with moderate to severe stable COPD according to the GOLD classifications (post-bronchodilator forced expiratory volume in 1 second (FEV_1_) ⩾30% but <80% of the predicted normal value, and post-bronchodilator FEV_1_/forced vital capacity <0.7).^18^ Patients must have been naive to both inhaler devices for ⩾2 years. Key exclusion criteria included the presence of clinically significant, uncontrolled chronic diseases (particularly those affecting coordination and/or motor system and other chronic respiratory diseases) and/or a COPD exacerbation within 6 weeks of Visit 1, or within 3 months if hospitalisation was required.

**Figure 1:**
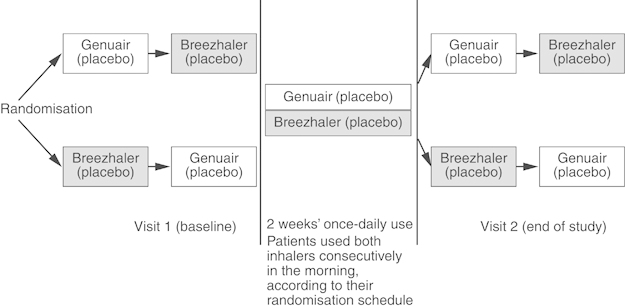
Study design.

The study consisted of two scheduled visits (Visits 1 and 2) with a 2-week period in-between (Figure 1). At Visit 1, patients were randomised (1:1) using a computer-generated randomisation schedule to inhale placebo via both Genuair and Breezhaler in one of two treatment sequences: Genuair first, Breezhaler second or Breezhaler first, Genuair second. At this visit, trainers demonstrated to patients the correct use of both inhalers according to the sequence in which the patient was instructed to use them. Following the demonstration, patients read the device instructions and demonstrated their use of the device until successful; a maximum of five attempts were permitted. Patients who failed to use either inhaler correctly after five attempts were discontinued from the study. After Visit 1, patients used both inhaler devices, containing placebo, once daily for 2 weeks. After 2 weeks (Visit 2), the trainers assessed patients’ preference, satisfaction and correct use of both inhalers, as well as their willingness to continue with each device and compliance with the inhaler devices. To assess inhaler technique, without further training, patients demonstrated use of both inhalers (in the same order as Visit 1) until successful, with a maximum of five attempts with each inhaler.

Patients continued with their usual medications (including those for COPD) throughout the study; this could include use of inhalers other than those being investigated in the study. The Genuair and Breezhaler devices contained only the lactose carrier (placebo).

### Assessments

Patients completed the multi-item Patient Satisfaction and Preference Questionnaire (PASAPQ)^19^ to measure their satisfaction and preference for both inhalers. This is a self-administered, 16-item measure of respiratory inhalation device satisfaction and preference for patients with asthma and COPD. It includes 13 satisfaction items measured on a Likert-type response scale from 1 (very dissatisfied) to 7 (very satisfied); these are grouped into two domains (performance and convenience) and constitute the total score (Supplementary Table 1). Each individual item contributes equally to the total score. The PASAPQ also contains an overall satisfaction question (measured on the 1–7 scale, with a score of 7 representing the highest level of satisfaction), a preference item (selection between devices or ‘no preference’) and a question on willingness to continue using the device (measured on a scale of 0 (not willing) to 100 (definitely willing)). During validation of the PASAPQ, the threshold for clinical relevance was estimated (the minimally important difference, MID). To achieve a small or medium effect difference in the performance domain required a difference of 4 or 10 points, respectively. For both the convenience domain and total score, the small or medium effect difference was estimated to be 3 or 8 points, respectively.^19^

The correct use of the inhaler devices was assessed by evaluating errors made by patients at Visits 1 and 2. Errors were categorised as critical or non-critical and were dependent on the individual inhaler (Supplementary Table 2). Critical errors were defined as those that compromised the potential benefit of the treatment, such as impeding drug deposition in the lungs or the delivery of an insufficient dose, while non-critical errors did not compromise the potential benefit of the treatment.

At Visit 2, patients’ compliance was assessed using the dose counter for Genuair and the number of capsules used and unused for Breezhaler (Supplementary Material). Compliance was defined as 10 out of 14 doses used.

The primary efficacy end point was the proportion of patients who preferred Genuair versus Breezhaler overall at Visit 2. The secondary end points were overall satisfaction with the inhaler device and critical inhaler technique errors at Visit 2. Additional efficacy variables included satisfaction with each of the individual performance and convenience attributes and willingness to continue using each inhaler.

### Statistical analyses

Patients’ preference for Genuair at Visit 2 was analysed using the Mainland–Gart’s test. In addition, a sensitivity analysis using Prescott’s test was carried out, which also included all patients who indicated ‘no preference’ for either inhaler. Mean PASAPQ total score, performance score, convenience score and score for individual attributes of each inhaler were analysed using an analysis of variance model for cross-over designs, including sequence, period and inhaler type as fixed factors and patient within sequence as a random effect. The differences between devices (estimated by differences between least square means) and standard error (s.e.) are also presented. The proportion of patients making ⩾1 critical error using each inhaler device at Visit 2 was analysed using Prescott’s test. All analyses were performed on the intent-to-treat population (all randomised patients who used both inhaler devices at least once and answered the preference question of the PASAPQ).

A minimum sample size of 120 patients was required to have 90% power to detect a preference of 65 versus 35% in favour of one of the inhaler devices (assuming 5% of patients expressed no preference or discontinued the study), with a two-sided test at the 5% significance level.

## Results

### Patients

**Figure 2:**
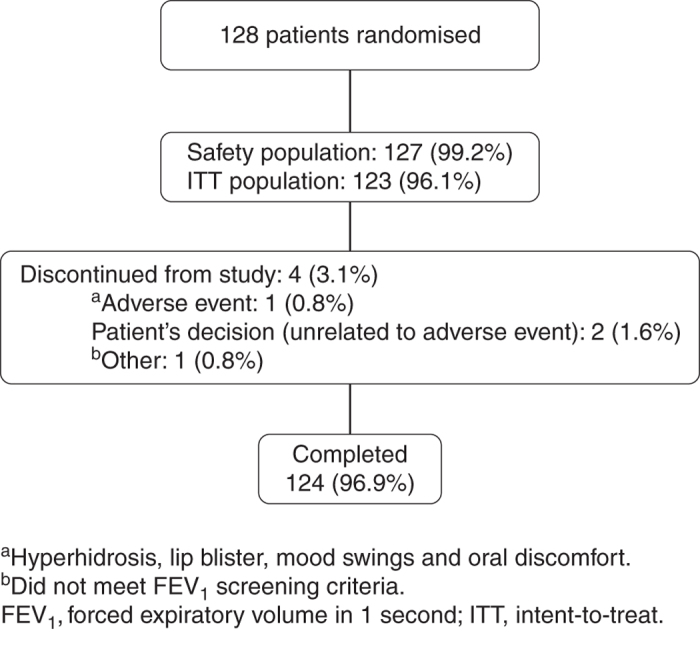
Patient disposition.

A total of 128 patients were enrolled and randomised in the study. Of these, 127 patients were included in the safety population (all randomised patients who used both inhaler devices at least once) and 123 in the intent-to-treat population; 124 patients completed the study (Figure 2). No patient was discontinued from the study for failing to demonstrate correct use of the study inhaler devices after five attempts at Visit 1. Of the 128 patients randomised, 98 (76.6%) and 96 (75.0%) achieved correct use of the Genuair and Breezhaler inhalers, respectively, at the first attempt at Visit 1; for both inhalers, five attempts were required for 100% of patients to achieve correct inhaler technique.

Demographics and baseline characteristics for the safety population are shown in Table 1. The majority of patients were male (*n*=91 (71.7%)); mean age was 67.6 years and mean predicted FEV_1_ was 49.3%. The most commonly used concomitant inhaler device was the HandiHaler, used by 75% of patients (Table 2). Overall compliance was equally high for both Genuair and Breezhaler (95.3 vs. 94.5%, respectively; Supplementary Material).

### Device preference

110 of the 123 patients in the intent-to-treat population who indicated a preference for either inhaler after 2 weeks, a statistically significantly higher proportion preferred Genuair to Breezhaler (72.7 vs. 27.3%, respectively; *P*<0.001). Sensitivity analysis using Prescott’s test to assess the influence of patients who indicated ‘no preference’ (13 patients, 10.6%) was consistent in showing that more patients preferred Genuair to Breezhaler (65.0 vs. 24.4%, respectively; *P*<0.001).

### Device satisfaction

**Figure 3:**
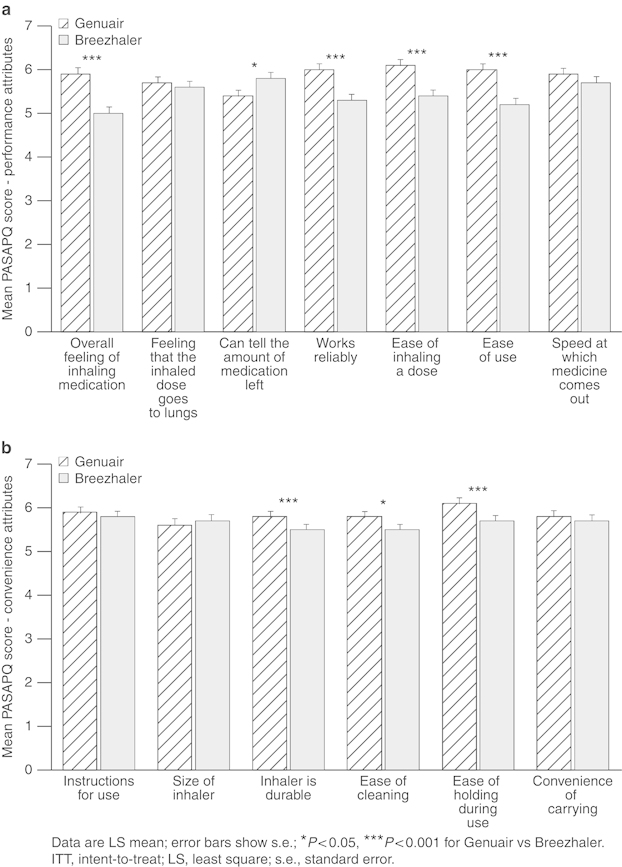
Mean satisfaction scores for attributes in the performance (**a**) and convenience (**b**) domains of the PASAPQ after two weeks of daily practice (intent-to-treat population; *N*=123).

The overall satisfaction score for Genuair at Visit 2 was statistically significantly greater compared with Breezhaler (mean (s.e.), 5.9 (0.15) vs. 5.3 (0.15), respectively; *P*<0.001). When the individual attributes of each inhaler were considered, patients were statistically significantly more satisfied with Genuair compared with Breezhaler for five of the seven performance attributes (Figure 3a) and for three of the six convenience attributes (Figure 3b).

Genuair was also statistically superior to Breezhaler in terms of patient satisfaction measured by total score (mean (s.e.), 80.5 (1.73) vs. 75.5 (1.73), respectively; *P*=0.002), overall performance score (80.5 (1.95) vs. 73.7 (1.95), respectively; *P*=0.002) and convenience score (80.6 (1.70) vs. 77.4 (1.70), respectively; *P*=0.022) of the PASAPQ. The MIDs between the inhalers in total score (5 points) and performance score (6.8 points) exceeded the small effect difference but did not achieve the medium effect difference. The difference in the convenience score (3.2 points) represented a small effect difference.

When patients were asked to indicate their willingness to continue using each inhaler, on a scale of 0–100, the mean score was statistically significantly greater for Genuair than Breezhaler (mean (s.e.), 79.6 (2.60) vs. 63.6 (2.60), respectively; *P*<0.0001), indicating a greater willingness to continue using Genuair. The mean difference in score (s.e.) between Genuair and Breezhaler was 16.0 (3.68; *P*<0.0001).

### Critical errors

After 2 weeks' use of Genuair and Breezhaler, fewer patients made critical errors at Visit 2, compared with Visit 1 (Visit 1, *n*=28 (22.8%) and *n*=30 (24.4%); Visit 2, *n*=4 (3.3%) and *n*=9 (7.3%), respectively). At Visit 2, there was no statistically significant difference in the number of patients who made ⩾1 critical inhaler technique error with only Genuair or only Breezhaler.

At Visit 2, 15 patients made a total of 41 inhaler technique errors, 31 of which occurred at the first attempt. The numbers of errors at each stage of inhaler use are shown in Table 3. At Visit 2, the most common critical errors (⩾2 patients) occurring on the first attempt with Breezhaler were: not inhaling strongly enough to hear the buzzing that indicates the capsule is spinning around the chamber (*n*=4 (3.3%)); the capsule not spinning around in the chamber when inhaling (*n*=2 (1.6%)); and not repeating the inhalation due to powder residue in the capsule and removal of the capsule immediately without checking for the presence of powder residue (*n*=2 (1.6%)). One patient made errors in all three of these steps of Breezhaler use. The only critical error made by ⩾2 patients on the first attempt of Visit 2 with Genuair was not exhaling before introducing the mouthpiece into the mouth (*n*=2 (1.6%)).

## Discussion

### Main findings

This randomised, cross-over study was designed to assess patients’ preference for overall satisfaction with and correct use of Genuair compared with Breezhaler after 2 weeks of daily use. The primary end point showed that significantly more patients with COPD preferred Genuair over Breezhaler. Furthermore, overall satisfaction was statistically significantly higher and patients were more willing to continue using Genuair than Breezhaler. No statistically significant difference was found between Genuair and Breezhaler in the proportion of patients who made critical inhalation technique errors during use.

### Interpretation of findings in relation to previously published work

The PASAPQ has been validated in patients with asthma, COPD and in patients with overlapping features of asthma and COPD.^19^ Post-validation, the questionnaire has been used in other inhaler comparison studies including a 12-week assessment of preference for the Respimat Soft Mist inhaler versus a multidose, prefilled, DPI in patients with asthma.^20^ In the study, both inhalers contained budesonide, and results revealed a preference for Respimat over the DPI. In a 48-week study comparing patient satisfaction using the Respimat inhaler and a metered-dose inhaler, both containing ipratropium bromide/albuterol in patients with COPD, the PASAPQ performance score was significantly higher with the Respimat inhaler and performance score MIDs were maintained at all treatment visits.^21^

Inhaler attributes rated as being ‘very important’ by patients with COPD are those associated with simplicity and ease of use.^3,22^ In the van der Palen^3^ study, among 105 patients with COPD, satisfaction with Genuair was significantly superior to the HandiHaler DPI device. In this study, although patients were not asked to rate the attributes of each inhaler, Genuair was statistically superior to Breezhaler in terms of satisfaction, with a number of attributes associated with those aspects deemed to be important to patients, including ease of inhaling a dose, ease of using the inhaler and ease of holding during use.

In this study, it was not possible to compare patient satisfaction scores with measures of clinical outcomes because both inhalers contained placebo. However, previous real-world studies have shown significant correlations between patients’ satisfaction with their inhaler and reduced exacerbation frequency, improved treatment compliance and improved health-related quality of life in patients with both asthma and COPD.^8,23^ Furthermore, during validation of the PASAPQ, Kozma *et al.* ^19^ proposed thresholds above which differences between the devices could be considered clinically meaningful (small and medium effect differences). In this study, Genuair was statistically superior to Breezhaler in terms of patient satisfaction measured by total score, and performance and convenience scores, with differences between the inhalers in convenience score (3.2 points) representing a small effect difference and differences in total score (5 points) and performance score (6.8 points) exceeding the small effect difference. Kozma *et al.* ^19^ acknowledge the challenges inherent in applying the concept of an MID to satisfaction measures given that they are more dependent on the devices being investigated and the population being studied than is the case with quality-of-life measures such as the EQ-5D.^19^ The higher level of patient satisfaction for Genuair over Breezhaler reported in this study may lead to improved clinical outcomes for patients using Genuair compared with Breezhaler in a real-world setting. We acknowledge, however, that in this 2-week study, compliance, which is also associated with improved clinical outcomes,^8,23^ was equally high for both inhalers.

Evidence suggests that up to half of patients using inhalers have errors in their inhalation technique and that most patients may not have their technique monitored by a clinician.^9,24^ The overall incidence of critical errors reported in this study was low for both inhalers. The number of critical errors with Genuair was similar to that previously reported in a randomised, cross-over study comparing Genuair with HandiHaler.^3^ Breezhaler and HandiHaler have a similar mechanism of action (capsule piercing system) and therefore a similar number of errors might be expected when assessing the two inhalers. Although there was a numerically lower incidence of critical errors with Genuair than Breezhaler in this study, there was no statistically significant difference between Genuair and Breezhaler in the number of critical errors at Visit 2. This result differs from the significantly lower incidence of errors previously reported for Genuair compared with HandiHaler.^3^ However, a high proportion of patients (75%) were using HandiHaler for the administration of concomitant COPD medication during our current study. Therefore, although there are a greater number of potential errors associated with Breezhaler compared with Genuair (due to the greater number of steps in the inhalation process), familiarity with HandiHaler may have improved Breezhaler technique and resulted in no overall difference in the number of errors observed compared with Genuair. The most common critical errors reported for Breezhaler (not inhaling strongly enough; the capsule not spinning during inhalation; and not repeating inhalation or checking for powder residue in the capsule), occurring in six patients in total, would be anticipated to mean the patient did not receive the intended dose of medication, potentially leading to reduced disease control.

### Strengths and limitations of this study

A strength of the present study is that the inhaler devices contained placebo rather than an active ingredient, while patients continued with their normal COPD medication. This approach minimises bias and allows a true reflection of device preference without the influence of treatment on patients’ airflow obstruction. During validation of the PASAPQ, attempts were made to estimate thresholds above which differences between inhalers could be considered clinically relevant (the MID). It remains to be determined whether the concept of an MID is relevant to a study comparing two inhalers, both containing placebo. It was also encouraging that more patients had an overall preference for Genuair compared with Breezhaler, regardless of whether patients responded with ‘no preference’. Further strengths of this study are that it was conducted over a wide geographical area, by a range of health care professionals from different backgrounds, and that assessments were made after 2 weeks of home use, which reflects real-life conditions of inhaler training and use. However, it is acknowledged that the time interval between training and assessment was relatively short. The 2-week time interval was considered sufficient to evaluate preference and correct inhaler use while also minimising inhaler burden given that patients continued their usual medications (including those for COPD) throughout the study. As inhaler errors occurred in this study despite the short time interval, the potential for error rate increasing over time should be investigated in the future. It is also acknowledged that the sequential use of different inhaler devices may not reflect real-life clinical practice. Genuair is currently used for the administration of aclidinium bromide, or aclidinium bromide plus formoterol fumarate, which requires twice-daily administration.^15–17^ However, given the high level of patient satisfaction with Genuair and willingness to continue use reported in this study, twice-daily use of this inhaler is unlikely to affect compliance.

### Implications for future research, policy and practice

The findings of this study indicate a preference for Genuair over Breezhaler among patients with COPD and provide a useful basis for further understanding of patient satisfaction with Genuair. Further investigation of the nature of critical errors may expand our knowledge of ways to improve patient inhaler technique. This is particularly pertinent in light of evidence suggesting that helping patients manage their condition and gain the maximum benefit from their inhaler may improve clinical outcomes.^4^ A study comparing Genuair with other inhalers over a longer time period may be useful to assess patient preference, satisfaction and compliance and evaluate the incidence of errors over the long term. It would also be interesting to evaluate patient satisfaction and compliance with Genuair in relation to clinical outcomes.

### Conclusions

In this study of patients with COPD, preference, satisfaction and willingness to continue using the inhaler were statistically significantly higher with Genuair compared with Breezhaler. The proportion of patients who made critical errors was low with both inhalers. Ensuring that patients are able to use their inhaler device correctly is likely to be important for increasing patient satisfaction and, consequently, improving treatment compliance and clinical outcomes in COPD.

## Figures and Tables

**Table 1:** Demographics and baseline characteristics (safety population)

*Variable*	*N*=127
Age, mean (s.d.), years	67.6 (8.0)
Gender male, *n* (%)	91 (71.7)
Duration of COPD, mean (s.d.), years	8.5 (8.1)
Predicted FEV_1_,^a^ mean (s.d.), %	49.3 (13.0)

Abbreviations: COPD, chronic obstructive pulmonary disease; FEV_1_, forced expiratory volume in 1 second; s.d., standard deviation.

a *N*=126.

**Table 2:** Concomitant inhaler device use during the study (safety population)

*Inhaler*, n* (%)*	*Genuair* */Breezhaler* (*N*=64)	*Breezhaler* */Genuair* (*N*=63)	*Total* *N*=127
Turbuhaler	23 (35.9)	17 (27.0)	40 (31.5)
Diskus	17 (26.6)	13 (20.6)	30 (23.6)
Respimat	8 (12.5)	7 (11.1)	15 (11.8)
pMDI	26 (40.6)	37 (58.7)	63 (49.6)
HandiHaler	48 (75.0)	47 (74.6)	95 (74.8)
Others	21 (32.8)	21 (33.3)	42 (33.1)

Abbreviation: pMDI, pressurised metered-dose inhaler.

**Table 3:** Number of inhaler technique errors at Visit 2, by error category^a^ (ITT population; *N*=123)

*Error category*	*Genuair*	*Breezhaler*
Critical errors prior to inhalation	0	0
Critical errors preparing for inhalation	2	3
Critical errors during inhalation	5	10
Critical errors after inhalation	NA	3
Non-critical errors	14	4

Some patients may have made errors with both inhalers and >1 error within each error category.

Abbreviations: ITT, intent-to-treat; NA, not applicable.

a Full list of errors within each category can be found in Supplementary Table 2.

## Notes

ADS has received speaker bureau fees, support for attending symposia, for consulting and for organising educational events from AstraZeneca, Bayer, Boehringer Ingelheim, Chiesi, Forest, GlaxoSmithKline, Novartis and TEVA. He has received institutional grant support for COPD research from AstraZeneca and for bronchiectasis research from Bayer, Forest, Gilead and Novartis. JH has received reimbursements for attending symposia, fees for speaking, for consulting and for organising educational events and funds for research from Almirall, AstraZeneca, Boehringer Ingelheim, Chiesi, Cipla, GlaxoSmithKline, Merck Sharp & Dohme, Mundipharma, Novartis and Teva. LP is an employee of Almirall. BS and AR are former employees of Almirall, and current employees of AstraZeneca. LR is an employee of Forest Research Institute, a subsidiary of Actavis PLC. HC has received sponsorship to carry out studies, consultant agreements and honoraria for presentations from Almirall, AstraZeneca, Boehringer Ingelheim, GlaxoSmithKline, Innovata Biomed, Meda, Napp Pharmaceuticals, Orion, Teva, Truddell Medical International and UCB. Research sponsorship has also been received from EPSRC and the MRC. The remaining authors declare no conflict of interest.
