# Supplementary material for: Preference, satisfaction and critical errors with Genuair and Breezhaler inhalers in patients with COPD: a randomised, cross-over, multicentre study
Source: NPJ Prim Care Respir Med. 2015 Apr 30;25:15018–. doi: 10.1038/npjpcrm.2015.18 (PMC4415437; doi:10.1038/npjpcrm.2015.18)
Supplement: Supplementary Table 1 [file npjpcrm201518-s1.pdf]

Supplementary Table 1. Items in the performance and convenience domains of the Patient Satisfaction and Preference Questionnaire

Device attribute	Question
**Performance**	Overall feeling of inhaling your medicine
	Feeling that inhaled dose goes to lungs
	Can tell amount of medication left in inhaler
	Works reliably
	Ease of inhaling a dose
	Ease of using the inhaler
	Speed medicine comes out
**Convenience**	Instructions for use
	Size of inhaler
	Durability of inhaler
	Ease of cleaning inhaler
	Ease of holding during use
	Convenience of carrying
