# Supplementary material for: Preference, satisfaction and critical errors with Genuair and Breezhaler inhalers in patients with COPD: a randomised, cross-over, multicentre study
Source: NPJ Prim Care Respir Med. 2015 Apr 30;25:15018–. doi: 10.1038/npjpcrm.2015.18 (PMC4415437; doi:10.1038/npjpcrm.2015.18)
Supplement: Supplementary Table 2 [file npjpcrm201518-s2.pdf]

Supplementary Table 2. Critical and non-critical errors for the Genuair® and Breezhaler® inhalers

**Genuair®**	**Breezhaler®**
**Critical errors prior to inhalation**	
Unable to open pouch with scissors	Unable to pull cap off
Mouth cap not removed before use	Unable to open mouthpiece
	Unable to remove capsule from the blister
	Second capsule exposed to light in the process of opening first capsule and exposed capsule not discarded
**Critical errors preparing for inhalation**	
Inhaler not held horizontally with green button facing upwards for priming the dose (45° tolerance)	Capsule swallowed instead of inhaling via inhaler
Inhaler shaken with mouthpiece facing the ground after priming the dose	Capsule placed directly into mouthpiece instead of into capsule chamber
Control window not green before inhalation	Damaged or contaminated capsule placed into capsule chamber
Mouthpiece facing downwards after preparation of dose	Mouthpiece not closed correctly before piercing the capsule
	Inhaler not held upright before piercing capsule
	Inhaler not held upright after capsule was pierced

	Mouthpiece facing downwards after preparation of dose
	Buttons on both sides not pressed simultaneously (capsule not pierced completely)
	Buttons to pierce capsule pressed more than once
	Inhaler shaken after capsule has been pierced
**Critical errors during inhalation**	
Did not exhale before introducing mouthpiece into mouth	Did not exhale before introducing mouthpiece into mouth
Exhaled into inhaler when dose is loaded	Exhaled into inhaler (mouthpiece) when capsule is already placed into the chamber and mouthpiece is in patient's mouth
Inhaler put down with control window still showing green	Buttons not released during inhalation
Button pressed whilst inhaling	Inhaler not held with buttons facing left and right
Did not inhale sufficiently through the inhaler (control window does not change from green to red)	Air entry blocked by finger whilst inhaling
Inhalation immediately stopped upon hearing the click	Inhalation not sufficiently strong to hear the buzzing indicating that the capsule is spinning around the chamber
	Capsule does not spin around in the chamber when inhaling due to possible problem with either the inhaler or the capsule

**Critical errors after inhalation**	
	Inhalation not repeated due to powder residue in the capsule and capsule immediately removed without checking for existence of powder residue
	Capsule not removed after inhalation
**Non-critical errors**	
Unable to open pouch with notch	Attempt to open mouthpiece without removing cap
Breath not held after inhalation	Breath not held after inhalation
Cap not replaced	Inhaler not emptied (empty capsule not removed prior to inserting new capsule)
Coughed immediately after inhalation	Mouthpiece not closed correctly
	Coughed immediately after inhalation
	Cap not replaced after closing mouthpiece
