# Supplementary material for: Preference, satisfaction and critical errors with Genuair and Breezhaler inhalers in patients with COPD: a randomised, cross-over, multicentre study
Source: NPJ Prim Care Respir Med. 2015 Apr 30;25:15018–. doi: 10.1038/npjpcrm.2015.18 (PMC4415437; doi:10.1038/npjpcrm.2015.18)
Supplement: Supplementary Information [file npjpcrm201518-s3.pdf]

Supplementary information

Patient Compliance

Patient compliance with both inhaler devices during the 2-week practice period was assessed. With Breezhaler, patients were asked to retain all used Breezhaler capsules. At Visit 2, all inhaler devices and Breezhaler capsules (used and unused) were collected and counted. Breezhaler compliance was assessed by noting the number of capsules properly used, according to the case report form (CRF), and the number of unused capsules. To assess Genuair compliance, the number of doses inhaled by the patient (based on the dose indicator on the inhaler device) was noted from the CRF. The dose counter on Genuair does not change if an unsatisfactory inhalation manoeuvre is used. For both inhalers, patients were deemed to be compliant within each treatment period if they used each inhaler on 10 of the 14 days (>70%).

For patients in the safety population compliance with both inhalers was high (Genuair, 95.3%; Breezhaler 94.5%) with no statistically significant difference between the inhalers. Patients who used Genuair first followed by Breezhaler (compliance of 98.4% for both inhaler devices) had higher compliance for both inhalers than patients who used Breezhaler first (compliance of 92.1% for Genuair and 90.5% for Breezhaler).
